# Accelerated Mucopermeation and Penetration of the Polypeptide Antibiotic Colistin via Ionic Complexation with Polyphosphazene Carriers

**DOI:** 10.1021/acsomega.6c01416

**Published:** 2026-05-01

**Authors:** Pauline Stadler, Stefanie Kehrer, Paul Strasser, Cornelia Roschger, Brigitta Loretz, Ian Teasdale

**Affiliations:** † Institute of Polymer Chemistry, 27266 Johannes Kepler University Linz, Altenberger Strasse 69, 4040 Linz, Austria; ‡ University Clinic for Cardiac-, Vascular- and Thoracic Surgery, Medical Faculty, Johannes Kepler University Linz, Altenberger Strasse 69 and Krankenhausstrasse 5, 4040 Linz, Austria; § Helmholtz Center for Infection Research (HZI), Helmholtz Institute for Pharmaceutical Research Saarland (HIPS), 66123 Saarbrücken, Germany

## Abstract

The mucus barrier plays a vital protective role for nonkeratinized epithelia in the body, yet its complex structure poses a significant obstacle to effective noninvasive, topical drug delivery. Here, we synthesize chain-end-labeled poly­(α-glutamic acid) (PGA) and poly­[di­(carboxylatophenoxy)­phosphazene] (PCPP) and use an artificial pulmonary mucus to evaluate the mucopermeation of the polymers compared with the established mucus-penetrating polymer poly­(ethylene glycol) (PEG). The biodegradable polyacids PGA and PCPP exhibited mucus permeability similar to that of the biopersistent PEG, even at high polymer concentrations (up to 50 mg/mL). To enable drug loading, PGA and PCPP were further examined for their ability to electrostatically complex the antibiotic lipopeptide Colistin (Polymyxin E) at different polymer-to-Colistin ratios. Colistin binding studies in PBS confirmed effective encapsulation by both polymers with PCPP-Colistin complexes exhibiting the highest stability. Mucopermeation studies of the polymer-Colistin complexes revealed enhanced Colistin mucopermeation for both polyacids, with an enhanced effect observed for PCPP complexes. Furthermore, the broth microdilution assay revealed no differences in the minimum inhibitory concentrations (MICs) of Colistin, PGA-Colistin, or PCPP-Colistin. Overall, PCPP showed significantly superior performance due to its higher Colistin encapsulation efficiency, attributed to its unique chemical structure, thereby highlighting its potential as a biodegradable mucus-penetrating carrier for cationic therapeutic peptides.

## Graphical abstract

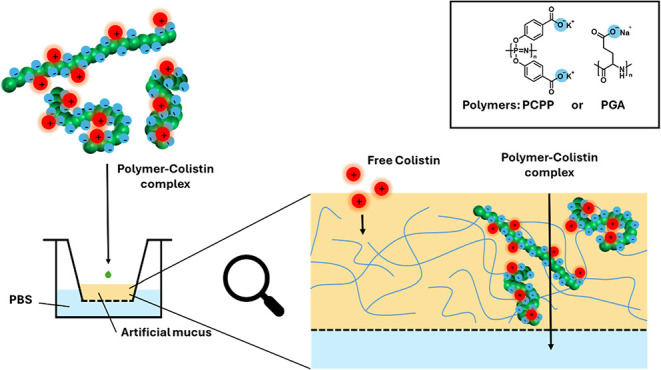

## 1 Introduction

The mucus layer acts as a protective barrier throughout the human body, including the eyes, respiratory tract, gastrointestinal tract, and reproductive organs. It shields the underlying epithelium from foreign materials and most pathogens while allowing small molecules, such as nutrients, to pass. The mucus is a viscous hydrogel composed mainly of water, mucin glycoproteins, nonmucin proteins, lipids, salts and cell debris.^1–3^ Its exact composition, thickness, pH, and other parameters vary with its location.^2^ The barrier properties do not only occur from its cross-linked and entangled structure, acting as a physical filtering barrier to trap particles via their size, but also via electrostatic interactions, hydrogen bonds, and disulfide bridges. Especially, its negatively charged glycoproteins lead to high interactions with cationic compounds, making it hard for them to pass.^1–3^ Additionally, the mucus layer is continuously renewed, and its thickness is balanced via the rate of secretion and degradation or shedding. Some toxic and irritating substances can stimulate mucus secretion and therefore increase the thickness of the mucus.^4^

The mucus barrier is often a challenge for topical mucosal drug delivery. In particular, for molecules at the upper limit of the Lipinski space, higher *M*_W_ and interaction potential require strategies for facilitated transport. They can be categorized in two opposite strategies: mucoadhesion and mucopenetration. In principle, both strategies are feasible; however, the optimal choice depends on the specific drug employed, the desired release profile (e.g., rapid versus sustained release), and the site of target mucus.^2^ In the case of mucoadhesion, the drug with its carrier sticks to the surface of the mucus to prolong the retention time and local release and therefore reduce the drug dosage. This is often realized with chitosan or thiolated polymers as carrier systems.^5,6^ Although this strategy shows promising results, it does not overcome the issue that certain cargos, such as tumor therapy agents, need to reach the epithelium and therefore need to penetrate the mucus layer first. Meanwhile in mucopenetrative drug delivery, the goal is to reduce interactions with the mucus components. Small, low-interacting carrier systems can diffuse into or through the mucus layer and reach the epithelium.^1,2^ A common strategy involves the use of poly­(ethylene glycol) (PEG) for encapsulation or as a surface coating of particles, called PEGylation. Its nonionic and hydrophilic nature minimizes adhesive interactions with mucus, thereby enabling passive mucopenetration. However, increasing the molecular weight of PEG can lead to steric entanglement with mucin fibers, thereby reducing its mucopenetration efficiency. In addition, high surface coverage of the particle is needed to ensure complete shielding from mucins.^1–3^

Indeed, PEG is one of the most commonly used polymers in all pharmaceutical applications. A major reason for this extensive use is its commercial availability in a broad range of molar masses and sufficient quality.^7^ PEG conjugates with drugs, proteins, and peptides show reduced systemic toxicity, enhanced stability, and *in vivo* circulation lifetime and improved pharmacokinetics.^8^ Although PEG is generally considered a safe option for applications in the medical sector, certain studies show complications when using PEG.^9^ A critical problem is the generation of anti-PEG antibodies by the immune system after repeated injection of a PEGylated nanomedicine. This leads to accelerated blood clearance and hypersensitivity reactions, as well as reduced therapeutic efficacy of the conjugated drug.^10^ Studies on tumor therapy show that the weak interaction of PEG with cancer cells hinders the uptake and the endosomal escape after endocytosis, overall reducing its therapeutic effect. Furthermore, PEG is a polyether, a structural feature that leads to high stability and therefore makes it difficult to degrade.^9^ These limitations of PEG highlight the urgent need for alternative polymers fulfilling the requirements of the biomedical field.

In this research, we will focus on two alternative polymers, poly­(sodium α-glutamic acid) (PGA) and poly­[di­(potassium carboxylatophenoxy)­phosphazene] (PCPP), and compare their mucopenetrating properties with those of PEG. Both polymers are highly water-soluble and widely reported to be biocompatible in various applications and biodegradable. Furthermore, their anionic nature as a polyacid makes them promising for mucopenetrative and permeative drug delivery.

In recent years, PGA has gained increasing attention in the field of polymer therapeutics. Several clinical studies with PGA-drug conjugates showed promising results. The PGA-paclitaxel conjugate Opaxio has been tested in clinical phase 3 trials via systemic application for the treatment of lung and ovarian carcinoma. The release kinetics of covalently bound cargos are governed by the degradation of the polymer, which in turn is mediated by the enzyme cathepsin B. The synthesis of the polypeptide is done via a ring-opening polymerization of N-carboxyanhydride (NCA), yielding narrow dispersities of 1.2–1.4.^7,11^

PCPP, a water-soluble member of the polyphosphazene family,^12^ is noted for its hydrolytic degradation and low cytotoxicity.^13^ PCPP’s potassium and sodium salts form noncovalent interactions with proteins, binding multiple proteins to create water-soluble complexes that transport, protect, and release payloads.^14^ Compared to other polyacids, PCPP is noted for its high density of binding sites (two per repeat unit) and the flexibility of its P–N backbone, which enhances protein binding.^15^ In extensive work pioneered by Andrianov and colleagues, PCPP has been shown to be particularly promising for its ability to stabilize antigens while presenting them to immune cells and was used with various antigens *in vitro*, *in vivo* (mice and large animals), and in human clinical trials.^16^ Shim et al. also showed that PCPP can be used as mucosal adjuvant for intranasal vaccination of mice against respiratory pathogens to enhance immunostimulation and function as an antigen delivery system.^17^ In this study, we perform a comparative study on the permeability of PGA, PCPP, and PEG of similar sizes through a mucus surrogate. We produced and characterized complexes of the peptide-antibiotic Colistin (Polymyxin E) and examined their mucus penetration and permeation. The objective is to investigate biodegradable polymeric carriers for facile peptide delivery into or across hydrogels, such as mucus, which could be extended to other cationic peptide antibiotics or peptide therapeutics that profit from topical mucosal delivery.

## 2 Results and Discussion

### 2.1 Polymer Synthesis and Characterization

A series of three water-soluble polymeric carriers, PCPP, PEG, and PGA, each with amine chain ends, were selected for this study (Figure 1), with their chain lengths adjusted to yield comparable hydrodynamic diameters of approximately 7–8 nm (Table 1 and Figure S1). While PEG was used as a mucopenetrating polymer reference, PCPP and PGA were chosen as suitable polymers for complexation with cationic peptides due to their negatively charged structure. The size of the polymers is small in relation to other particle forms, so they are expected to have lower entanglement in the mucin mesh. An α-amine-PCPP was synthesized via a living cationic polymerization using a styrene-functionalized initiator to control the length and α-chain-end of the polymer and facilitate site-specific labeling.^18,19^ Cysteamine hydrochloride was added to the vinyl group of the styrene via the thiol–ene addition reaction. After deprotection of the pendant propyl-esters with KOH, the α-amine group was used as an attachment point for labeling with cyanine-5 NHS-ester (scheme in Figure S2). Linear Cy5-labeled PGA was synthesized via a ring-opening polymerization of benzyl glutamic acid N-carboxyanhydride (NCA) according to a known literature procedure.^20^ A known challenge for this polymerization is the occurrence of side reactions, as end-group termination and chain transfer, which limit the formation of high molecular weight PGA.^21^ Consequently, the target *M*_W_ was calculated based on the initial amount of used NCA, but lower molecular weights were expected due to incomplete conversion and side reactions. Notably, the NMR shows higher *M*_W_ than those obtained by SEC measurement. This discrepancy may arise from shielding effects of the polymer backbone, that affect signal integration, as well as from uncertainties with integrating the low intensity initiator signal. PEG-bis-amine was purchased with a *M*_W_ of 20 kDa and labeled with cyanine-5 NHS-ester on the amine end-groups.

**Figure 1:**
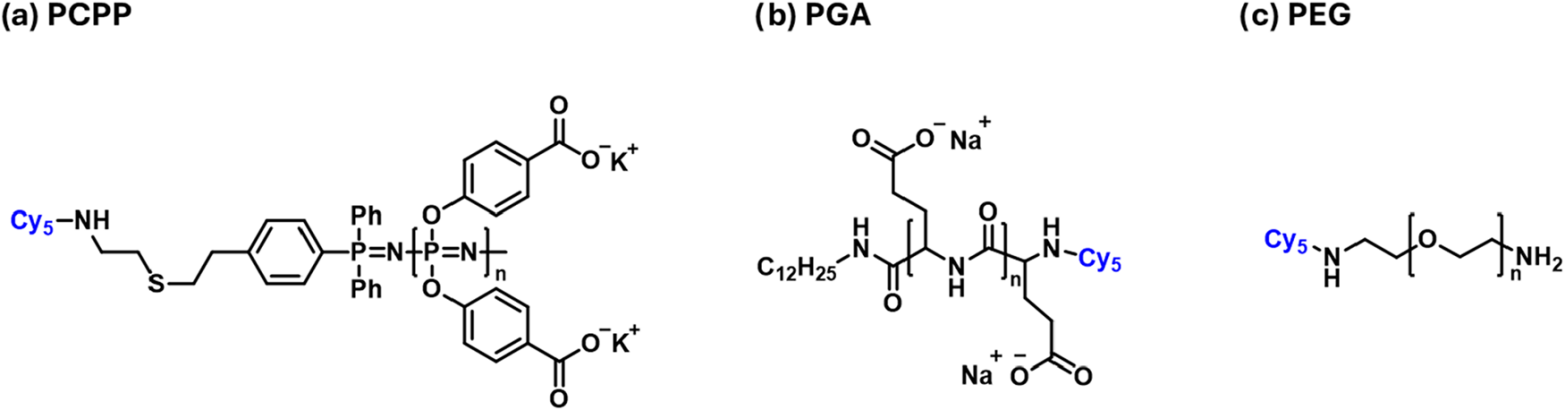
Chemical structures of the chain-end functionalized, cyanine-5 (Cy5) labeled polymers (a) poly­[di­(carboxylatophenoxy)­phosphazene] (PCPP), (b) poly­(α-glutamic acid) (PGA), and (c) poly­(ethylenglycol) (PEG).

**Table 1:** Molecular Weight (Target, Calculated from NMR Spectroscopy and from SEC Analysis), *Đ* (from SEC Analysis) and *d*_h_ (Hydrodynamic Diameter Peak Average) from DLS Measurement of the Polymers

polymer	*M*_W_ target, kDa	*M*_W_ NMR, kDa	*M*_W_ SEC, kDa	*Đ*	*d*_h_, nm
PCPP	39.5	37.5	38.9^b^	1.151^b^	8.0^b^
PGA	60.4	37.3	17.0^b^	1.06^b^	7.3^b^
PEG	20^a^				7.5^b^

a Supplier data.

b Measurement of a different batch without Cy5-labeling

### 2.2 Polymer-Colistin Complexes

Polymer complexes of PGA and PCPP were then formed with the cationic cyclopeptide Colistin via electrostatic binding, as illustrated in Figure 2. Colistin (Polymyxin E) belongs to the class of Polymyxin antibiotics and is a cationic and cyclic polypeptide, used as an antimicrobial against Gram-negative bacteria. It is one of the few approved antibiotics for inhalation therapy of pulmonary infections.^22^ Size measurements by volume distribution of the polymer-Colistin complexes in PBS, as shown in Figure 3, only show a signal at a size comparable to the free polymer, while some larger aggregates are visible in the raw intensity plots (Figures S3 and S4). No signal corresponding to free Colistin (H_2_O) or Colistin aggregates (PBS) could be detected. This may be either from complete complexation of Colistin or due to over-representation of the larger species in the intensity signal. Furthermore, the ζ-potential of the complexes (10:1) in water is only slightly shifted to higher values for PGA-Colistin (from −73.2 to −66.8 mV) and PCPP-Colistin (from −55.1 to −53.3 mV), indicating efficient encapsulation of Colistin.

**Figure 2:**
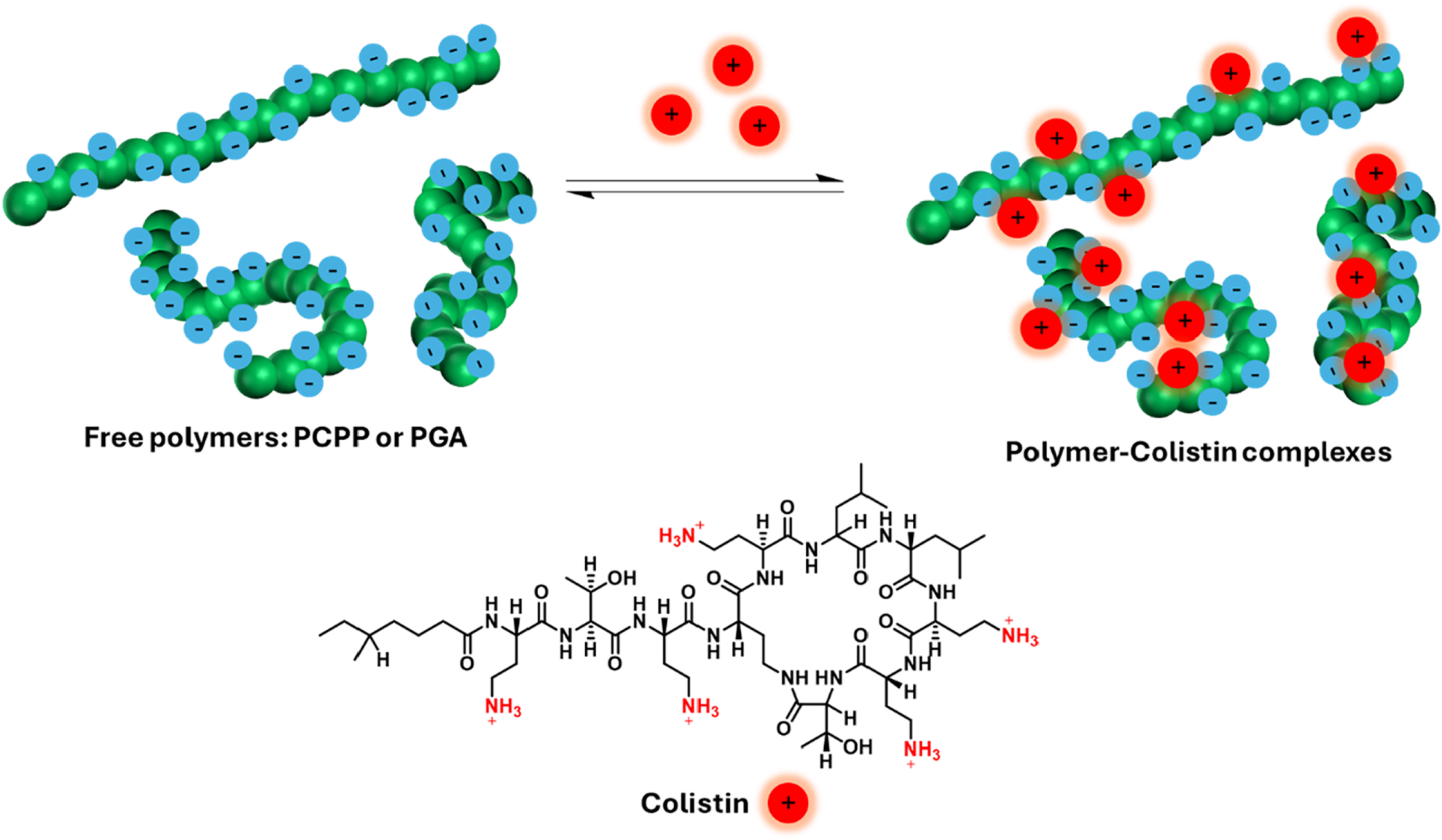
Schematic illustration of electrostatic binding between polymers and Colistin & chemical structure of Colistin.

**Figure 3:**
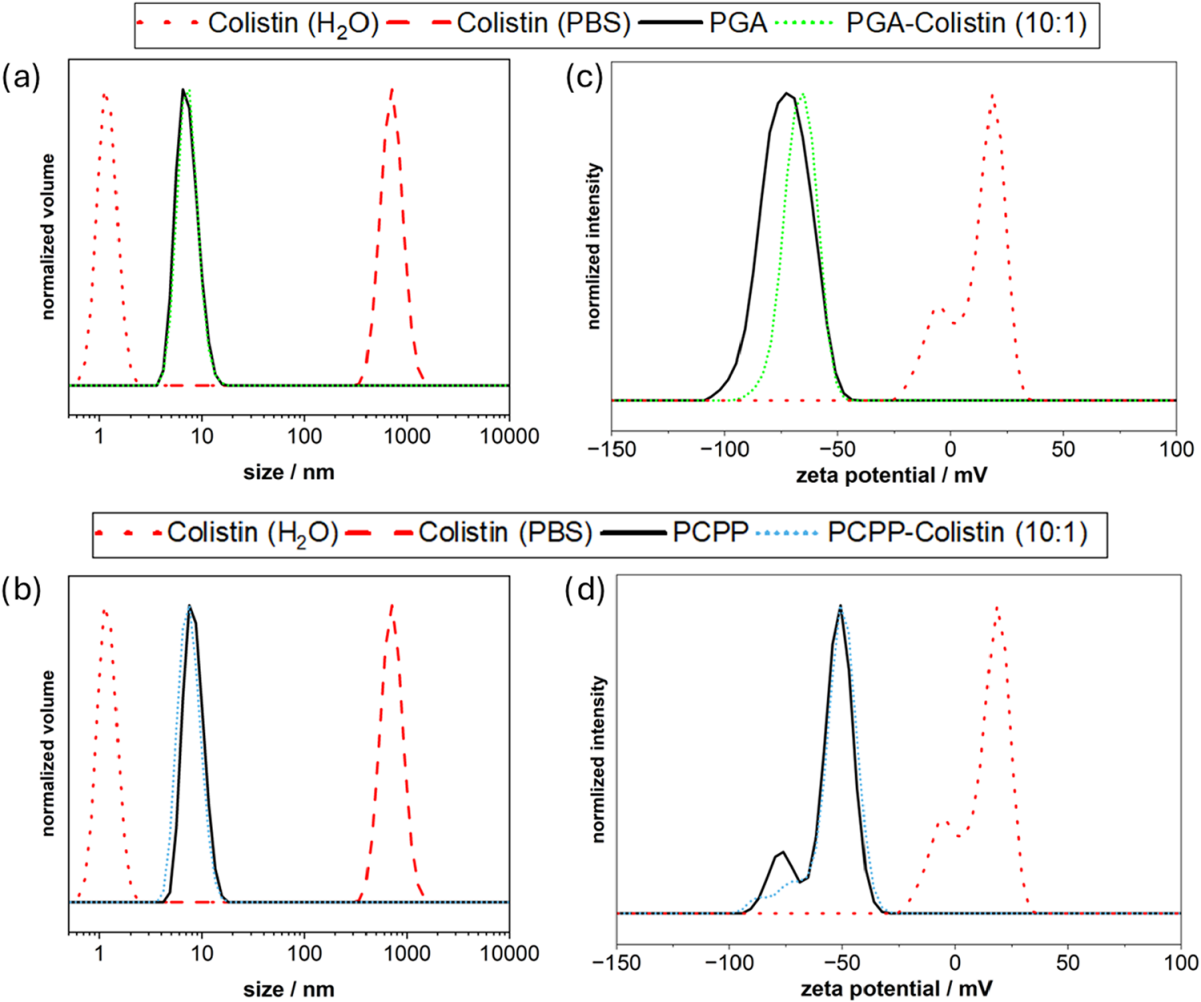
DLS size measurement of (a) PGA-Colistin complexes and (b) PCPP-Colistin complexes in PBS pH 7.4 and ζ-potential of (c) PGA-Colistin complexes and (d) PCPP-Colistin complexes in Milli-Q-water in a polymer-to-Colistin ratio of 10:1.

### 2.3 Polymer-Colistin Complex Stability Studies

The stability of the polymer-Colistin complexes was evaluated via dialysis and detection of free Colistin outside of the membrane via fluorescence measurements of the FITC-label. Therefore, polymer-to-Colistin ratios of 10:1 (Figure 4) and 5:1 (Figure S5) were tested in PBS buffer, pH 7.4, at room temperature. Both polymers were capable of effectively binding Colistin at these ratios. Higher Colistin loading may still be possible for both polymers but can lead to increased particle size due to aggregation and lower stability of the formed complexes. Notable differences in stability were observed, with PCPP forming more stable complexes than PGA. After 8 h, the PCPP-Colistin complex at a 5:1 ratio released only 5% of its Colistin loading under these conditions, whereas the corresponding PGA conjugate released 21%. This trend continued over longer incubation times and became even more pronounced in comparison to free Colistin. After 24 h, Colistin release from PCPP remained minimal at 11%, in contrast to 26% released from PGA. These results indicate a superior encapsulation efficiency of PCPP that may arise from a low barrier to rotation and the unique flexibility of the P–N backbone,^23^ the higher density of carboxylic acid groups or its amphiphilic nature, or indeed a combination of these features.^24^ The influence of the temperature on the release of Colistin from the complexes (10:1 ratio) was further examined, where room temperature and 37 °C were compared (Figure S6). As expected, a slightly faster release was detected for free Colistin and PGA-Colistin, as well as a very minor increase in the level of PCPP-Colistin.

**Figure 4:**
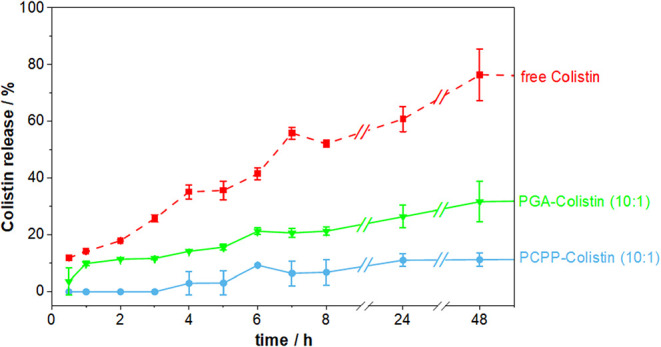
Colistin release from Polymer-Colistin complexes at a ratio of 10:1 (w/w%).

Although the complex exhibits high stability under physiological conditions, the ionic nature of the interaction permits cargo release in biological environments, where local pH fluctuations or competitive binding events can effectively disrupt the electrostatic encapsulation.

### 2.4 Mucopermeation of Polymers

Artificial pulmonary mucus was chosen as a model to compare the mucopermeation and penetration of the polymers. Poly­(acrylic)­acid (PAA) was added to the mucus to obtain comparable mechanical properties as in native mucus, as already described by Huck et al.^25^ While artificial pulmonary mucus provides a controlled model for evaluating transport, it does not fully replicate the structural complexity of native mucus. As highlighted by Huck et al.,^25^ differences in cross-linking density may influence molecular diffusion, and thus validation in native mucus systems is warranted. In this study, a mucus layer thickness of 2 mm was used to provide optimal conditions for assessing the mucopermeating behavior and for comparing the performance of the polymers. We used PEG as a reference polymer due to its well-known mucopenetrating properties. Two distinct sample concentrations were tested for each polymer to evaluate the effect of the concentration. As shown in Figure 5, the polymers exhibit mucopermeating properties comparable to those of PEG, reaching mucopermeation above 50% after 3 h. No concentration-dependent effects were observed over the range of 25–50 mg/mL, suggesting that permeation through the mucus layer is primarily diffusion-limited and remains effective at high concentrations.

**Figure 5:**
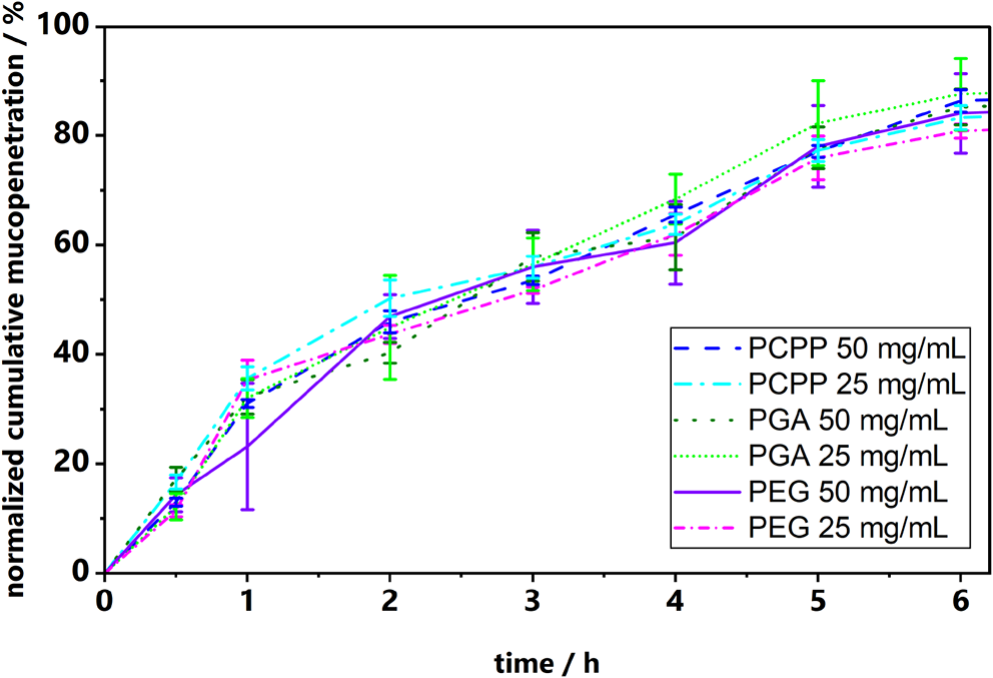
Mucopermeation of the free polymers poly­(α-glutamic acid) (PGA), poly­[di­(carboxylatophenoxy)­phosphazene] (PCPP), and poly­(ethylene glycol) (PEG) at 50 and 25 mg/mL (maximum fluorescence of the respective polymer stock was set to 100% after 24 h).

### 2.5 Mucopermeation of Colistin

The mucopenetrative properties of Colistin are generally considered to be poor due to its strong positive charge and high affinity for mucin and other anionic components of mucus, as discussed in the literature. Huang et al.^26^ demonstrated that Colistin can bind to mucin, which may be the reason for reduced antibacterial activity when administered via pulmonary delivery. Frisch et al.^27^ reported that Colistin permeation at a concentration of 0.6 mg/mL across native mucus and artificial pulmonary mucus did not show significant differences with generally low permeation (<5%). Although the surrogate overestimates the actual barrier properties of mucus, it can be used as a suitable model for Colistin permeation. We found that the transport across artificial pulmonary mucus may be dose-limited for Colistin (Figure S7). We evaluated two concentrations, 2.5 and 5 mg/mL, and observed no increase in mucopermeation at the higher concentration during the first 4 h. Thus, a 2-fold increase in Colistin concentration did not result in a proportional increase in permeation. This supports the hypothesis that the mucus hinders permeation of Colistin. Overall, Colistin exhibited slower permeation across the mucus layer than the polymers, indicating that the process is not solely diffusion-driven but is also affected by additional hindering interactions. Potential electrostatic interactions between the cationic Colistin and the anionic mucus matrix may contribute, in addition to steric hindrance associated with micelle formation at elevated Colistin concentrations.^23^

### 2.6 Mucopermeation of Polymer-Colistin Complexes

We hypothesized that transport of Colistin across the mucus layer can be improved by forming complexes with PCPP and PGA via electrostatic interactions between the cationic amine groups of Colistin and the anionic carboxylic acid groups of the polymers, thereby encapsulating the antibiotic. Two different formulations were tested with polymer-to-Colistin ratios of 10:1 and 5:1. At lower Colistin content (10:1 ratio), illustrated in Figure 6, only minor differences in the transport across the mucus of PGA-Colistin complexes in relation to free Colistin were observed. However, PCPP-Colistin complexes show a distinct increase in Colistin transport, leading to a 10-fold higher mucopermeation within 30 min, and 1.6-fold after 3 h. Figure 7 shows the mucopermeation at a higher Colistin loading (5:1 ratio). After 3 h, the PGA complex enhanced Colistin mucopermeation 1.8-fold, whereas the PCPP complex enhanced colistin mucopermeation by 2.4-fold. These findings indicate that, while both polymers enhance the mucopermeation of Colistin, PCPP showed significantly higher increase, presumably due to the superior encapsulation efficiency (vida supra). In general, the effects of the polymers are less intense at lower concentrations, where the dose-limiting effect of Colistin transport may be less pronounced. A physical PEG-Colistin mixture was tested as an additional reference, although PEG cannot electrostatically bind Colistin, due to its neutral charge (Figure S8). A nonsignificant increase of PEG-Colistin mucopermeation may indicate that minor encapsulation of Colistin with PEG occurs.

**Figure 6:**
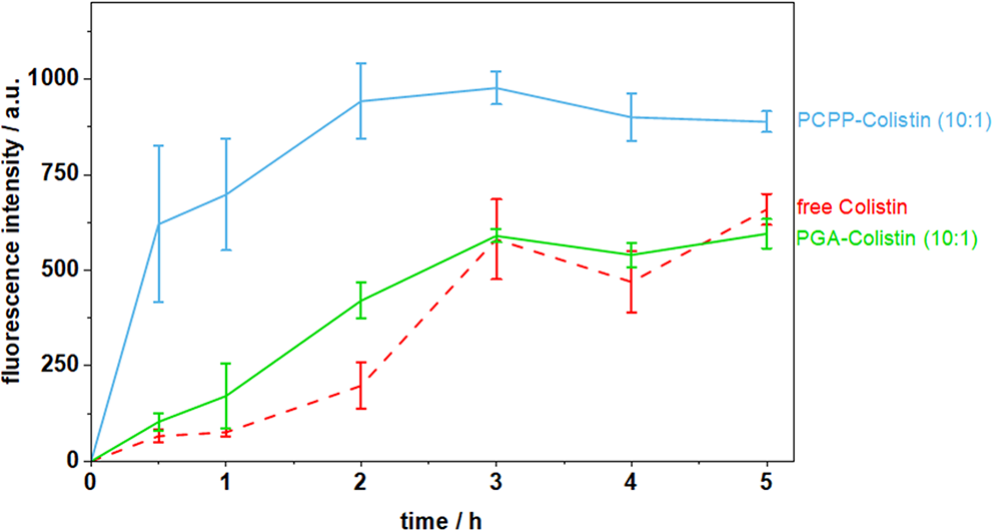
Mucopermeation of Polymer-Colistin complexes in a ratio of 10:1 (25:2.5 mg/mL) and free Colistin at comparable concentration (2.5 mg/mL).

**Figure 7:**
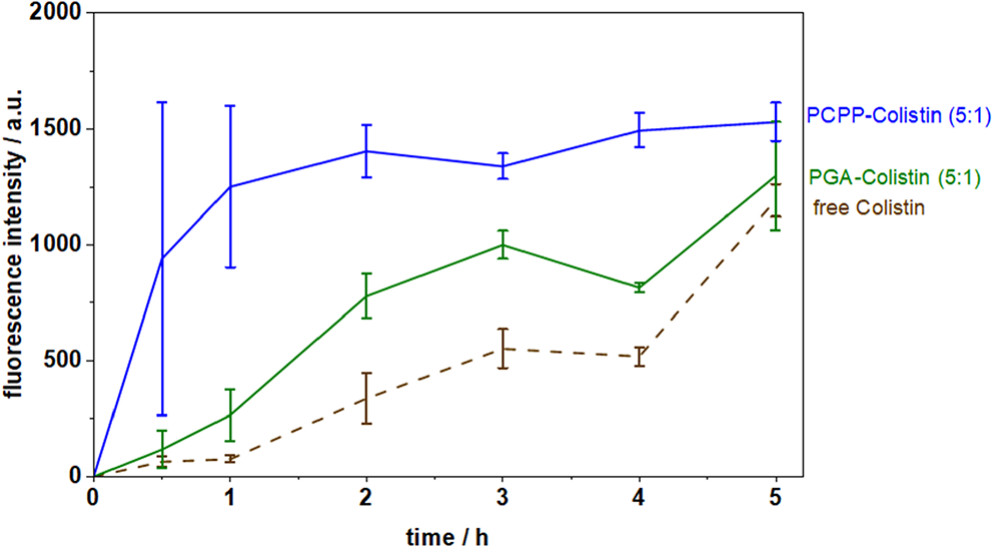
Mucopermeation of Polymer-Colistin complexes in a ratio of 5:1 (25:5 mg/mL) and free Colistin at a comparable concentration (5 mg/mL).

### 2.7 Antibacterial Activity

To ensure that conjugation of PGA or PCPP to Colistin does not alter its antibacterial activity, the broth microdilution method, the gold standard for Colistin, was performed using the *E. coli* K12 strain. The polymers alone showed no effect on the bacteria. Colistin, PGA-Colistin, and PCPP-Colistin all exhibited a minimum inhibitory concentration of 1 μg/mL at both polymer-Colistin ratios (10:1 and 5:1). These results indicate that the polymers do not interfere with the antibacterial efficacy of Colistin (Figures 8 and S9).

**Figure 8:**
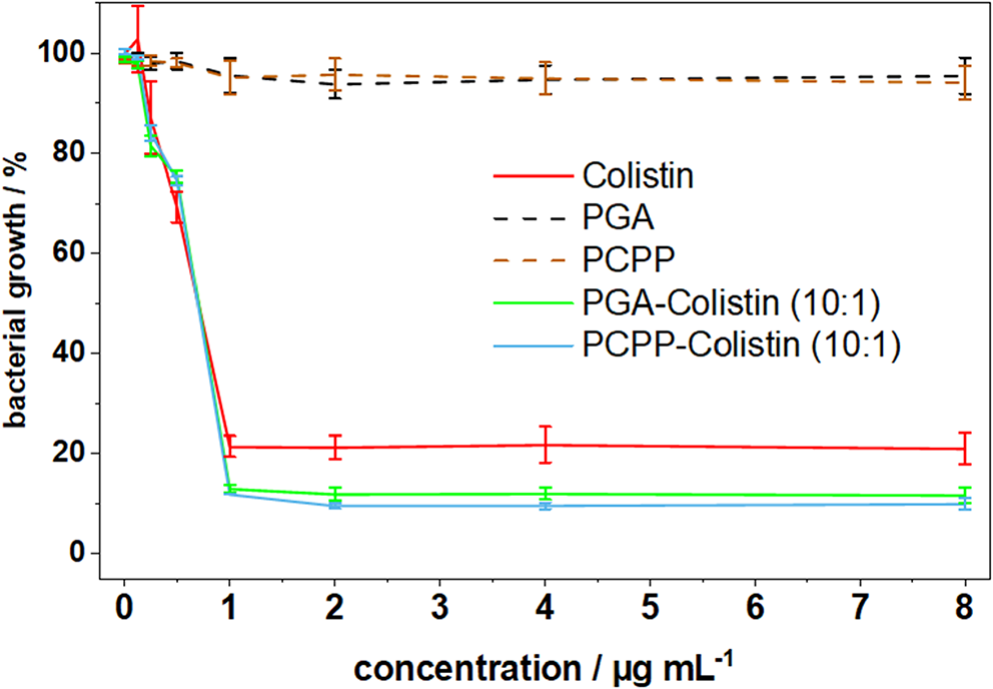
Antibacterial activity of polymer–Colistin conjugates (10:1), compared with free polymers and free Colistin, tested on strain *E. coli* K12. Polymer concentrations were adjusted to ratios of 10:1 relative to the corresponding Colistin concentration.

## 3 Materials and Methods

Chemicals were obtained from different suppliers and used as purchased unless otherwise specified. Propyl 4-hydroxybenzoate sodium salt, 4-(diphenylphosphino)­styrene, hexachloroethane, cysteamine hydrochloride, mucin from porcine stomach (type II), desoxyribonucleic acid (low-molecular weight from salmon sperm), poly­(acrylic)­acid (Carbopol 974P NF), egg yolk emulsion (Oxoid), casein hydrolysate, diethylenetriaminepentaacetic acid, and trizma base were purchased from Sigma-Aldrich, Cyanine-5 NHS-Ester (Cy5) from Lumiprobe, potassium hydroxide (KOH), sodium hydroxide (NaOH), and sodium chloride (NaCl) were purchased from Fisher, and potassium chloride was obtained from J.T. Baker. Colistin sulfate (23202 u/mg), FITC isothiocyanate isomer I, PEG-bis­(amine), and (ethyl (2,4,6-trimethylbenzoyl) phenylphosphinate) (TPO-L) were bought from BLDpharm. Dimethylformamide (DMF) and tetrahydrofuran (THF) were purchased from Fisher, pyridine from Roth, anhydrous THF and cation-adjusted Mueller–Hinton broth from Thermo Scientific, and anhydrous dichloromethane (DCM) from Alfa Aesar. Deuterated solvents chloroform-*d* (CDCl_3_) and deuteriumoxide (D_2_O) were bought from Deutero. THF for dialysis was reused after distillation. *Escherichia coli* K12 strain (HB101) from BioRad and McFarland BSS 0.5 standard from Carl-Roth were used. Ultrapure water (Milli-Q-water) was obtained from a Millipore device. Dialysis membranes from Spectra/Por RC were used with suitable molecular weight cutoffs (MWCO). Samples were lyophilized with an Alpha 1–2 LDplus freeze-drying system from Christ. An argon-filled glovebox from MBraun was used for the synthesis of the polymers, as stated.

NMR measurements were recorded on a Bruker Avance III 300 MHz spectrometer. SEC of the intermediate products in organic media was performed on a Viscothek GPCmax instrument using a precolumn (300 mm × 8 mm, 10 μm particle size) and 3 GRAM Columns (300 mm × 8 mm, 10 μm particle size). The samples were filtered through 0.2 μm PTFE syringe filters prior to injection and eluted with DMF containing 10 mM LiBr as the mobile phase at a flow rate of 0.75 mL min^–1^ at 60 °C. The molecular weights were measured using a conventional calibration and multiple detector calibration of the light scattering, refractive index, and viscosity detectors calibrated with a polystyrene standard from Polymer Standard Service GmbH (PSS) with a d*n*/d*c* value of 0.1433 for poly­[bis­(propyl 4-hydroxy benzoate)­phosphazene]. SEC of the products in aqueous media was performed on an Agilent Technologies 1260 Infinity II system equipped with a Shodex OHpak LB-802.5 (300 × 8 mm^2^, 6 μm particle size) and a Shodex OHpak LB-804 (300 × 8 mm^2^, 10 μm particle size) column. Samples were filtered through 0.2 μm nylon syringe filters prior to injection, at a flow rate of 0.5 mL min^–1^, and detected via a UV–Vis detector from Agilent, a refractive index detector RI-501 from Shodex, and a TREOS II light scattering detector from Wyatt technology. PGA is eluted with Milli-Q water with 0.1 M NaNO_3_ and 0.025 wt % NaN_3_, while for PCPP, a PBS buffer pH 7.4 containing 0.025 wt % NaN_3_ is used. Verification of Cyanine-5 labeling was done via UV-detection at 600 nm. Molecular weights were calculated via a light scattering detector. Results were analyzed with ASTRA 8.3.0 from Wyatt with a d*n*/d*c* of 0.1675 for PGA^20^ in water and 0.272 for PCPP (sodium salt)^28^ in PBS. Size and ζ-potential measurements were performed on a Malvern Zetasizer Nano ZSP at 20 °C at a fixed scattering angle of 173°. The sample solutions (1 mg mL^–1^) were prepared in PBS buffer pH 7.4 for size and Milli-Q-water for the ζ-potential. Fluorescence intensity was recorded on a SpectraMax M2e Multi-Mode Microplate Reader with a set emission wavelength of 670 nm for Cy-5 and 520 nm for FITC. Bacterial growth was assessed using a Promega GloMax Microplate Reader at 600 nm.

SEC spectra can be found in Figures S10–S12, NMR spectra in Figures S13–S15, and size measurements in Figure S1.

### 3.1 Synthesis of Cyanine-5 Labeled PGA

Cyanine-5 labeled linear PGA was synthesized and labeled according to known literature procedures^20^ with dodecylammonium BF_4_ as an initiator and 400 equiv of 5-benzyl *L*-glutamate NCA was used for polymerization. Removal of the benzyl ester protection group was done with trimethylsilyl iodide (TMSI), resulting in a rest benzyl content of <3%.

### 3.2 Synthesis of Cyanine-5 Labeled PCPP

#### 3.2.1 Synthesis of Trichlorophosphoranimine (Cl_3_PNSiMe_3_)

The trichlorophosphoranimine monomer was synthesized according to known literature procedure.^20,29^

#### 3.2.2 Synthesis of Poly­[bis­(propyl 4-hydroxy benzoate)­phosphazene]

The synthesis was adapted from a literature procedure.^14^ Chlorination of the initiator and polymerization were performed in an argon-filled glovebox. Propyl 4-hydroxybenzoate sodium salt was dried at 80 °C for 24 h in a vacuum oven prior to use.

Briefly, 4-(diphenylphosphino)­styrene (20 mg, 0.069 mmol, 1 equiv) was dissolved in 0.5 mL of anhydrous DCM and combined with hexachloroethane (18 mg, 0.076 mmol, 1.1 equiv), dissolved in 0.5 mL of anhydrous DCM. The solution was stirred overnight, resulting in a chlorinated initiator species. For polymerization, the monomer trichlorophosphoranimine (1.557 g, 6.93 mmol, 100 equiv) was dissolved in little anhydrous DCM, added to the initiator, and stirred overnight. Macrosubstitution of the chlorine atoms was done by suspending propyl 4-hydroxybenzoate sodium salt (5.61 g, 27.7 mmol, 400 equiv) in approx. 50 mL of anhydrous THF and dropwise addition of the polydichlorophosphazene. The mixture was stirred overnight at room temperature in the glovebox and another 72 h at 55 °C under an argon atmosphere. Afterward, the solvent was evaporated under reduced pressure, and the polymer was precipitated three times into water and dialyzed (3.5 kDa MWCO) overnight in THF. Afterward, the solvent was removed under reduced pressure, yielding a clear solid. Yield: 1.2 g (43%); ^1^H NMR (CDCl_3_): 0.93 ppm (t, 607H, −C*H*_3_, *J* = 7.5 Hz), 1.67 ppm (sxt, 401H, −C*H*_2_–, *J* = 6 Hz), 4.12 ppm (t, 379H, −O–C*H*_2_–, *J* = 6 Hz), 5.42 ppm (d, 1H, styrene-CH=C*H*_2_, *J* = 9 Hz) 5.79 ppm (d, 1H, styrene–CH=C*H*_2_, *J* = 18 Hz), 6.50–6.90 ppm (br, 381H, Ar-*H*), 7.05–7.24 ppm (m, 14H, initiator), 7.38–7.73 ppm (br, 391H, Ar-*H*);^31^P NMR (CDCl_3_): −20.63 ppm, −16.48 ppm, 7.48 ppm of SEC (DMF): *M*_W_ (conventional calibration): 30.9 kDa, *Đ*: 1.2; *M*_W_ (multidetector calibration): 61.1 kDa, *Đ*: 1.1.

#### 3.2.3 α-Functionalization with Cysteamine

900 mg portion of poly­[bis­(propyl 4-hydroxy benzoate)­phosphazene] (0.022 mmol, 1 equiv) was dissolved in 3 mL of DMF. 20 mg of TPO-L (2.2 wt %) and 25 mg of cysteamine hydrochloride (0.22 mmol, 10 equiv) were added, the solution was degassed for 10 min, and stirred under 365 nm overnight at 5 °C. The reaction mixture was then dialyzed (3.5 kDa MWCO) in water and THF for 3 and 24 h, respectively. 560 mg (62%) of a clear solid was obtained. ^1^H NMR (CDCl_3_): 0.93 ppm (t, 607H, −C*H*_3_, *J* = 7.5 Hz), 1.67 ppm (sxt, 401H, −C*H*_2_–), 4.12 ppm (t, 379H, −O–C*H*_2_–, *J* = 6 Hz), 6.50–6.90 ppm (br, 381 H, Ar-*H*), 7.05–7.24 ppm (m, 14H, initiator), 7.38–7.73 ppm (br, 391H, Ar-*H*);^31^P NMR (CDCl_3_): −20.64 ppm, −16.48 ppm, 7.48 ppm.

#### 3.2.4 KOH Deprotection of the Ester

560 mg portion of the resulting polymer was dissolved in 5 mL of THF and 2.5 mL of 12 M KOH was added and vigorously stirred overnight. Afterward, the precipitate was filtered, redissolved in water, and dialyzed (3.5 kDa MWCO) against water for 24 h. Freeze-drying yielded a fluffy white polymer. Yield: 300 mg (55%); ^1^H NMR (D_2_O): 6.56 (br, 416H, Ar-*H*), 6.95–7.20 (m, 14H, Initiator), 7.35 (br, 421H, Ar-*H*); ^31^P NMR (D_2_O): −18.98 ppm, −14.16 ppm

#### 3.2.5 Cyanine-5 Conjugation to the α-Amine Functionalized PCPP

300 mg of PCPP (0.0075 mmol) was dissolved in 3 mL PBS-buffer pH 8.4 and the pH was readjusted using 0.1 M NaOH. 2.2 mg of Cyanine-5 NHS-ester (0.0033 mmol, 44 mol %) was added in 2 portions of 0.5 mL of PBS pH 8.4. The solution was stirred for 24 h in the dark and subsequently dialyzed (3.5 kDa MWCO) against Milli-Q-water for 72 h. Freeze-drying yielded a blue polymer.

### 3.3 Cyanine-5 Labeling of PEG-bis­(amine)

100 mg portion of PEG-bis­(amine) (0.005 mmol) was dissolved in 3 mL of PBS pH 8.2 and combined with 1.5 mg of Cyanine-5-NHS-ester (0.0022 mmol, 45 mol %) dissolved in 1.5 mL of PBS pH 8.2. The mixture was stirred overnight in the dark prior to dialysis (3.5 kDa MWCO) in water for 24 h. Freeze-drying yielded 96.8 mg of a blue solid.

### 3.4 FITC Labeling of Colistin Sulfate

Briefly, 100 mg of Colistin sulfate (0.072 mmol) was dissolved in 2 mL of water and combined with 1.5 mg of 5-FITC isothiocyanate (0.0036 mmol, 5 mol %) dissolved in 2 mL of pyridine. The mixture was stirred overnight in the dark. Afterward, 30 mL of water was added and freeze-dried. The solid orange product was washed two times with cold diethyl ether, three times with cold ethanol, and once with cold diethyl ether. The FITC labeling was verified via HPLC.

### 3.5 Synthesis of Artificial Pulmonary Mucus Surrogate

The artificial pulmonary mucus surrogate was synthesized according to known literature procedure.^25^

Briefly, 50 mg of mucin (type II, mucin from porcine stomach), 50 mg of desoxyribonucleic acid (low-molecular weight from salmon sperm), 50 mg of sodium chloride, 22 mg of potassium chloride, and 18 mg of trizma base were dissolved in 8 mL of Milli-Q-water. 100 μL of a 0.15 M diethylenetriaminepentaacetic acid was added, and the solution was stirred overnight to dissolve the mucins completely. Afterward, 50 mg of casein hydrolysate and 50 μL of egg yolk emulsion (Oxoid) were added, as well as 0.9% (w/v) poly­(acrylic)­acid to increase the viscoelastic properties of the mucus. The pH was adjusted to 7.0 by using 1 M NaOH.

### 3.6 Preparation of Polymer-Colistin Complexes

Polymer-Colistin formulations were prepared by mixing equal volumes of Cy5-labeled polymer stock solution (50 mg/mL in PBS) with FITC-labeled Colistin stock solutions of different concentrations to obtain the desired polymer-to-drug ratios. For a 10:1 polymer-to-Colistin ratio (w/w%), 0.5 mL of the polymer stock was combined with 0.5 mL of Colistin at 5 mg/mL in PBS. For a 5:1 ratio (w/w%), the polymer stock (0.5 and 50 mg/mL) was mixed with 0.5 mL of Colistin at 10 mg/mL in PBS. Free Colistin controls were prepared at matching Colistin concentrations. An additional control with PEG-Colistin was made analogous to those of the other polymer-Colistin complexes.

### 3.7 Polymer-Colistin Complex Stability Studies

For the release study, 150 μL of each formulation were diluted with 850 μL PBS and transferred into dialysis tubing (3.5 kDa MWCO), which was then placed in 15 mL of PBS. Samples were maintained on an orbital shaker at 200 rpm at room temperature. Quantification of the FITC-Colistin release was performed by fluorescence spectroscopy. At predetermined time points, 200 μL of the external buffer were collected and immediately replaced with an equal volume of fresh PBS. Three experiments per formulation were performed. Single experiments for comparing the effect of temperature were made at room temperature and 37 °C without shaking.

### 3.8 Mucopermeation Studies

Mucopermeation studies were adapted from literature procedure.^27^ Corning transwell inserts with an area of 0.9 cm^2^ and a 3.0 μm filter were filled with each 180 μL of artificial pulmonary mucus, leading to a layer thickness of 2 mm. The inserts were placed in a receptor compartment, filled with 800 μL PBS buffer pH 7.4. 20 μL of each sample were applied onto the artificial mucus. Samples for fluorescence measurements were taken (100 μL, diluted with 100 μL fresh PBS) from the receptor compartment and the volume was replaced with fresh PBS buffer. Three experiments per formulation were performed. Fluorescence intensity shows a linear correlation either with the amount of FITC-labeled Colistin or the amount of Cyanine-5 labeled polymers, dependent on the chosen wavelength.

### 3.9 Testing of Antibacterial Activity

The antibacterial activity of Colistin and the polymers PGA and PCPP was evaluated using the broth microdilution method with an *Escherichia coli* K12 strain. Bacterial cultures and inoculum preparation followed established microbiological guidelines. Cultures were suspended in cation-adjusted Mueller–Hinton broth (CAMHB) and adjusted to a density of 5 × 10^5^ CFU/mL. Colistin served as the reference antibiotic. The linear polymers PGA and PCPP were tested either alone or in combination with Colistin. Serial 2-fold dilutions of Colistin, ranging from 0.125 to 128 mg/L, were prepared for each test condition. Polymer concentrations were adjusted to ratios of 10:1 or 5:1 relative to the corresponding Colistin concentration. Assays were conducted in 96-well microtiter plates, with each well containing 100 μL of the test compound and 100 μL of the *E. coli* inoculum. Appropriate growth and medium controls were included. Plates were sealed and incubated at 37 °C for 24 h. Bacterial growth was quantified at 600 nm using a Promega GloMax Microplate Reader. The MIC was defined as the lowest concentration at which no visible growth occurred compared to the growth control.

## 4 Conclusions

This study demonstrates that the biodegradable polyacids PGA and PCPP exhibit mucopermeation capabilities comparable to those of the “gold-standard” PEG, highlighting their potential as alternative carriers. PGA and PCPP were subsequently tested as carriers for the cationic antibiotic Colistin (Polymyxin E), where two polymer-to-Colistin ratios (10:1 and 5:1) were evaluated. Both polymers effectively encapsulate Colistin, with PCPP forming the most stable complexes in PBS at pH 7.4. Furthermore, complexation with these polymers enhanced Colistin transport across the mucus layer with more pronounced effects observed at higher polymer-to-Colistin ratios. The superior mucopermeation of PCPP complexes is attributed to their higher encapsulation efficiency, highlighting PCPP as a promising biodegradable platform for the mucus-mediated delivery of cationic therapeutics, while preserving their antibacterial activity. This finding, together with PCPP’s excellent safety profile demonstrated in vaccine and protein studies, makes it a highly promising candidate for use as a pulmonary mucosal carrier for Colistin. However, given that artificial pulmonary mucus does not fully replicate native mucus, additional experimental evaluation is still required.^22^ Nevertheless, former transport studies across artificial and native mucus for Colistin^27^ already showed a similar behavior and the comparable behavior of PGA and PCPP to PEG, which is an established mucopenetrative polymer of similar size (7–8 nm), suggests a high likelihood that these polymers will maintain their performance in native mucus environments. For future translational application, it will also be essential to determine whether these polymer-Colistin formulations or even higher Colistin loadings are compatible with aerosol generation and stable under conditions relevant to pulmonary drug delivery. Together, these findings expand the toolbox of biodegradable anionic polymers suitable for overcoming mucus barriers and may support the development of improved therapies for pulmonary infections.

## Supplementary Material



## Notes

Dynamic light scattering (DLS) measurements; synthesis schemes; Colistin release profiles; additional mucopermeation data; antimicrobial activity data; SEC chromatograms; and NMR spectra (PDF)
