# Supplementary material for: Accelerated Mucopermeation and Penetration of the Polypeptide Antibiotic Colistin via Ionic Complexation with Polyphosphazene Carriers
Source: ACS Omega. 2026 May 1;11(18):27302–10. doi: 10.1021/acsomega.6c01416 (PMC13177036; doi:10.1021/acsomega.6c01416)
Supplement: Supplementary file 1 [file ao6c01416_si_001.pdf]

Supporting Information

Accelerated mucopermeation and -penetration of the polypeptide antibiotic Colistin via ionic complexation with polyphosphazene carriers

Pauline Stadler[1], Stefanie Kehrer[2], Paul Strasser[1], Cornelia Roschger[2], Brigitta Loretz[3] and Ian Teasdale[1]*

[1]Johannes Kepler University Linz, Institute of Polymer Chemistry. Altenberger Strasse 69, 4040 Linz, Austria, [2]Johannes Kepler University Linz, University Clinic for Cardiac-, Vascular- and Thoracic Surgery, Altenberger Strasse 69, 4040 Linz and Krankenhausstrasse 5, 4020 Linz, Austria, [3]Helmholtz Institute for Pharmaceutical Research Saarland (HIPS), Helmholtz Center for Infection Research (HZI), 66123 Saarbrücken, Germany

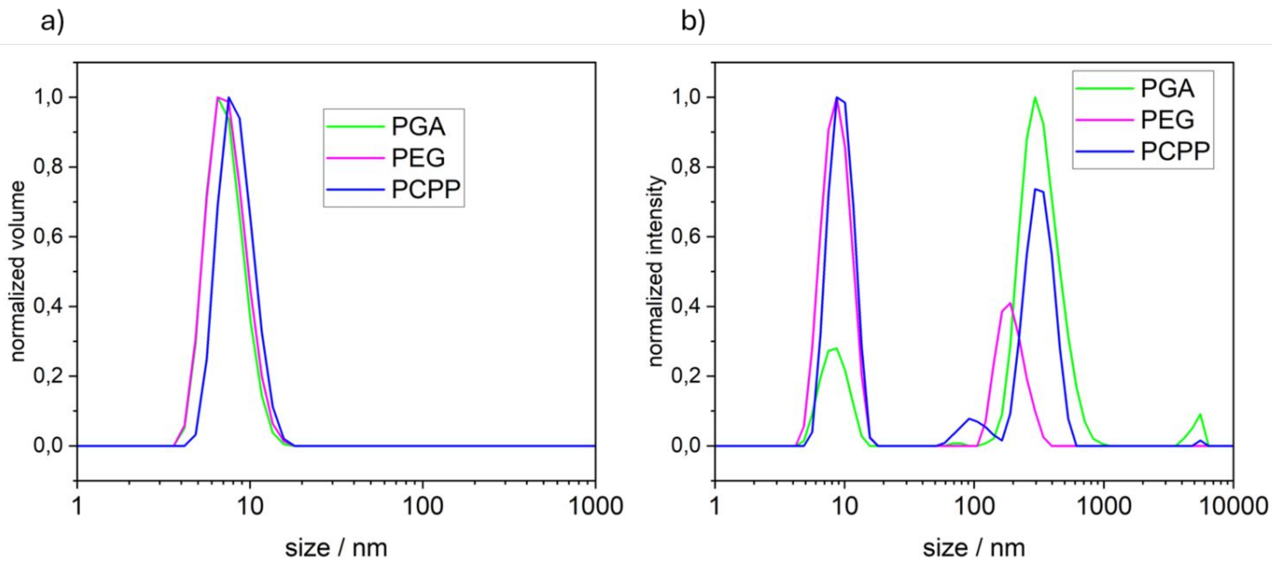

Figure S 1. DLS size measurement of the polymers (1 mg/mL) in PBS pH 7.4 by a) normalized volume distribution and b) normalized intensity distribution

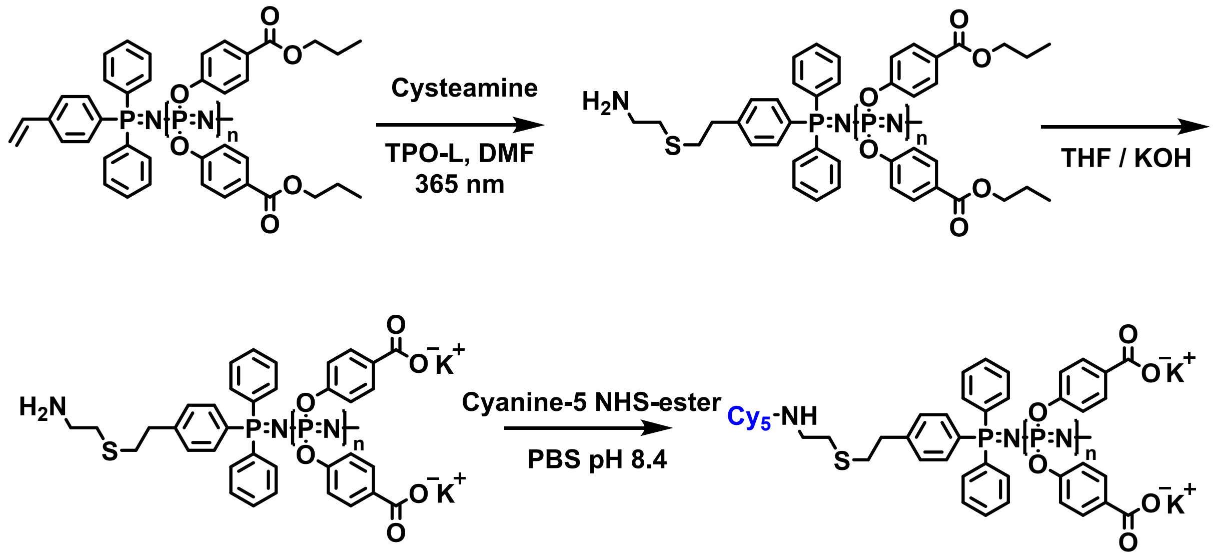

Figure S 2. Synthesis of Cyanine-5 labelled PCPP polymer, starting with the synthesis of poly[bis(propyl 4-hydroxy benzoate)phosphazene], continued with end-functionalization with cysteamine followed by deprotection of the ester with KOH und subsequent labelling with Cyanine-5.

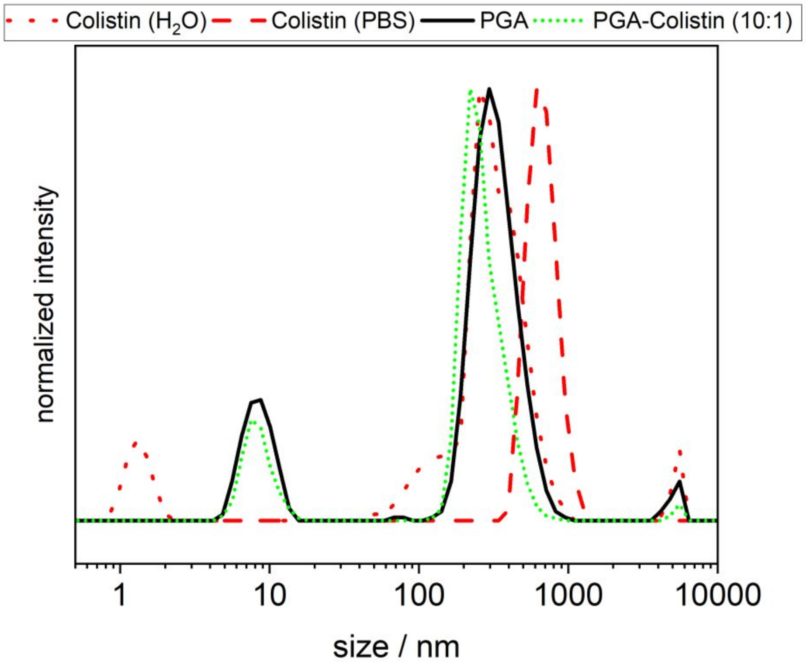

Figure S 3. DLS size measurement of PGA-Colistin complexes in PBS (normalized intensity).

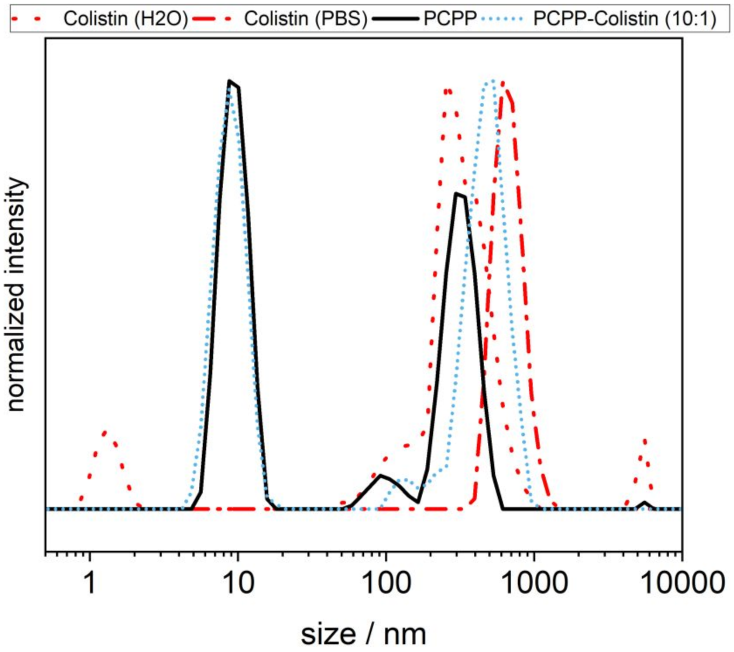

Figure S 4. DLS size measurement of PCPP-Colistin complexes in PBS (normalized intensity).

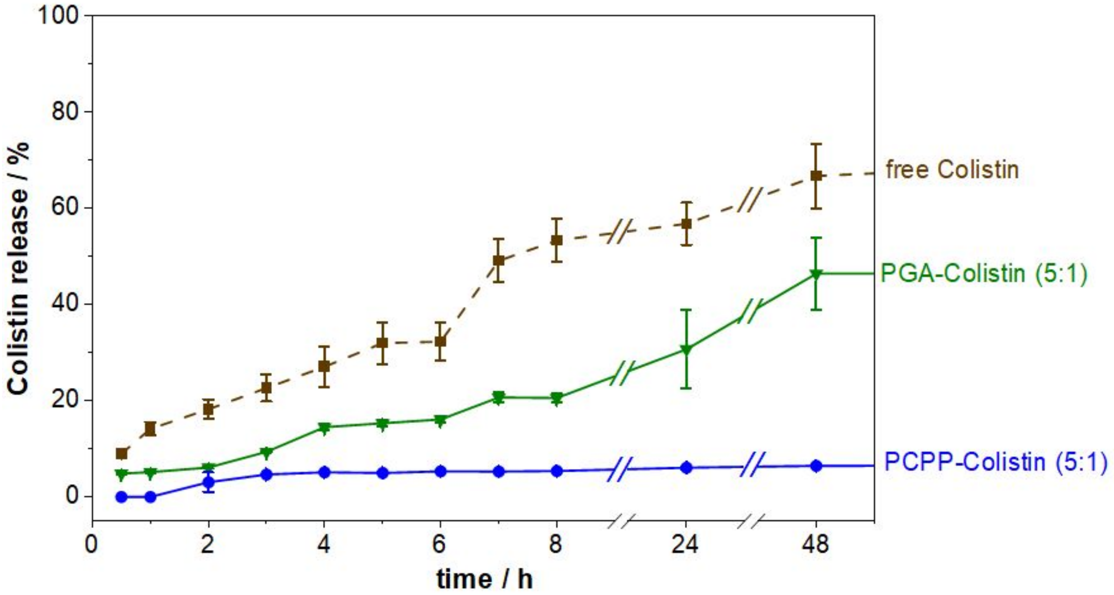

Figure S 5. Colistin release from Polymer-Colistin complexes at a ratio of 5:1 (w/w%) in PBS pH 7.4.

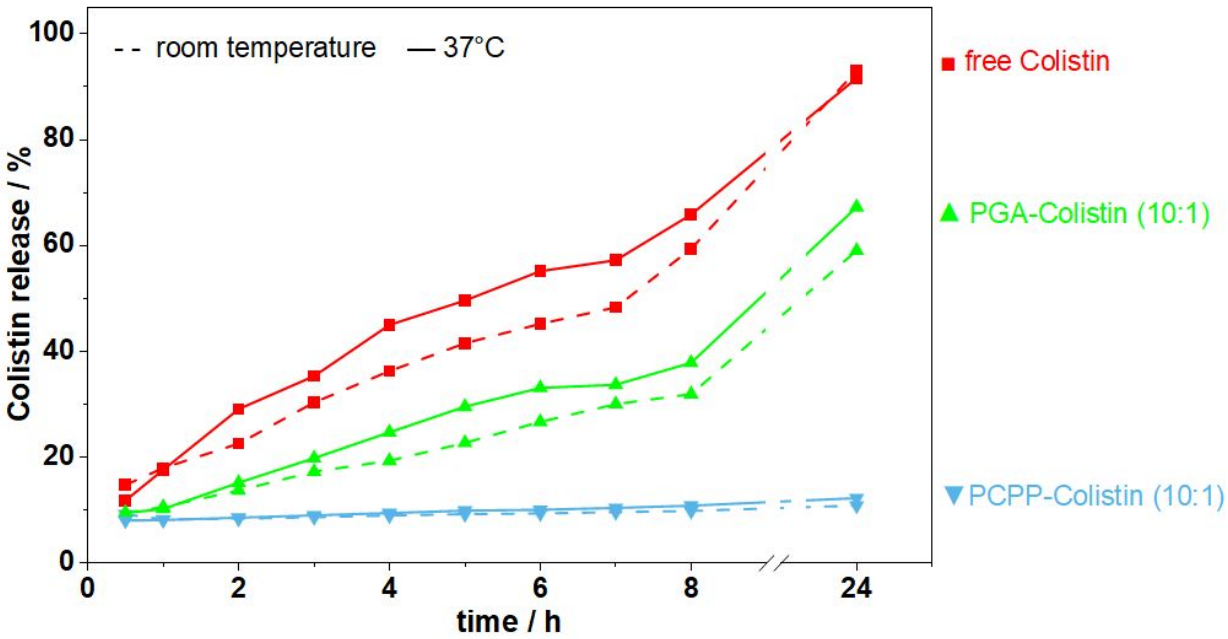

Figure S 6. Colistin release from Polymer-Colistin complexes at a ratio of 10:1 (w/w%) in PBS pH 7.4 at room temperature (dashed line) vs. 37˚C (continuous line).

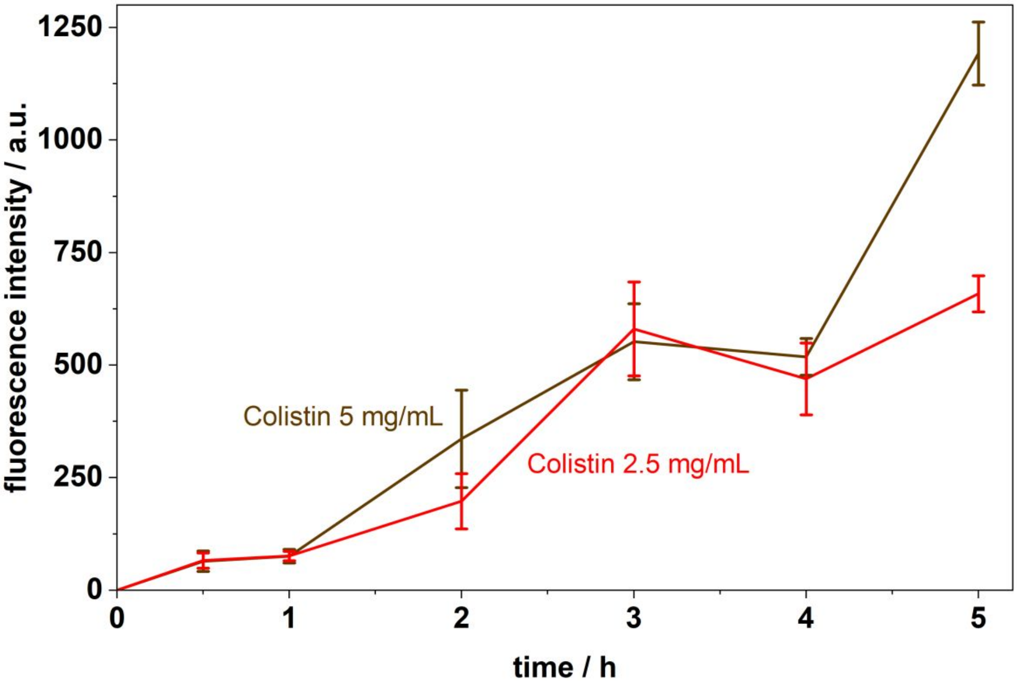

1.1.

Figure S 7. Mucopermeation of Colistin at 2.5 mg/mL and 5.0 mg/mL.

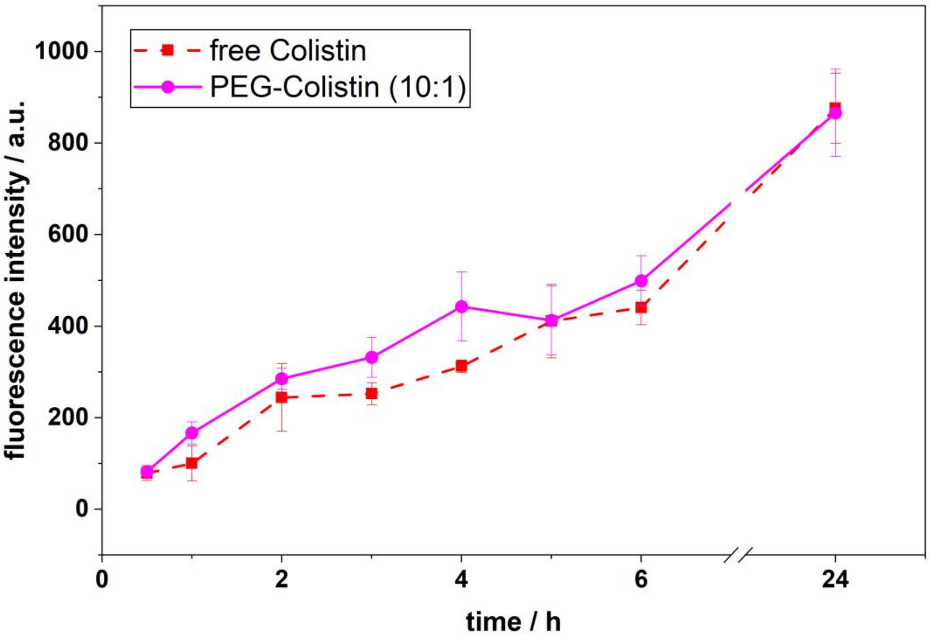

Figure S 8. Mucopermeation of PEG-Colistin mixture in a ratio of 10:1 (25:2.5 mg/mL) and free Colistin at comparable concentration (2.5 mg/mL).

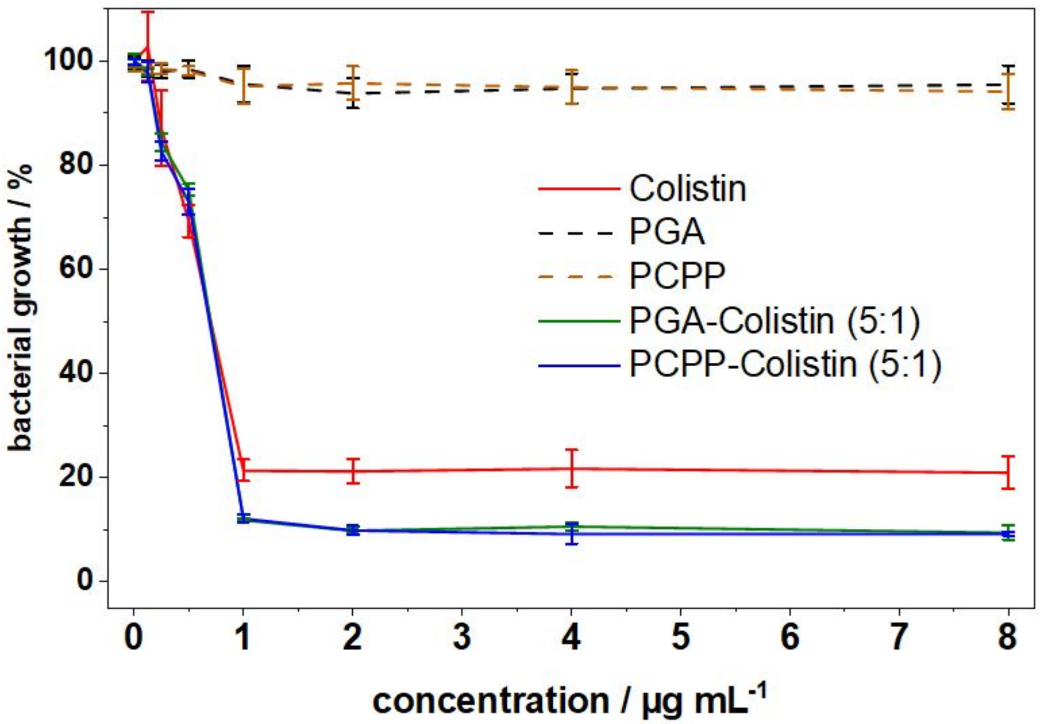

Figure S 9. Antimicrobial activity of Polymer–Colistin complexes (5:1), compared with free polymers and free Colistin, tested on strain E. coli K12. Polymer concentrations were adjusted to ratios of 5:1 relative to the corresponding Colistin concentration.

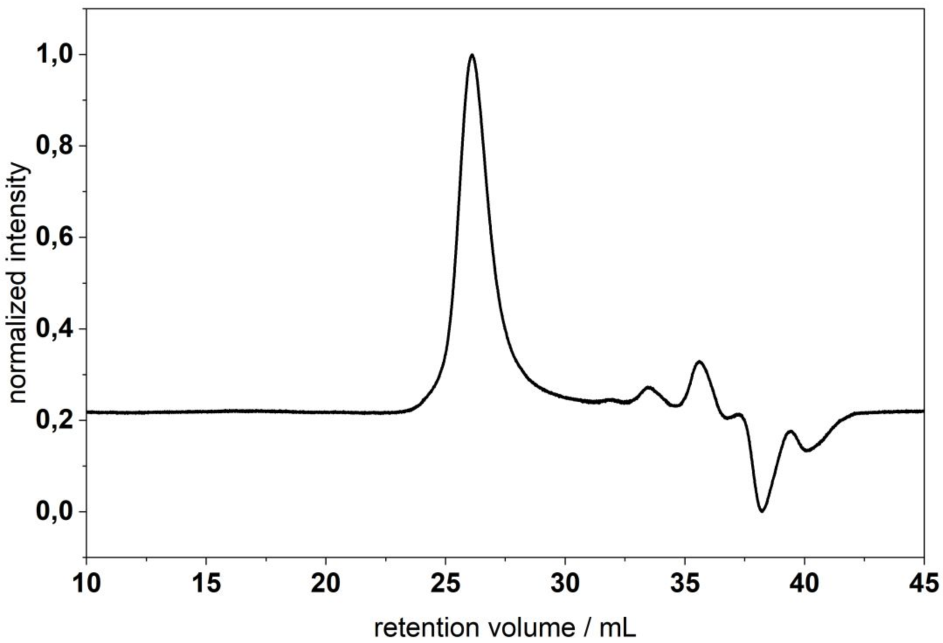

Figure S 10. SEC of poly[bis(propyl 4-hydroxy benzoate)phosphazene] in DMF (RI detector response).

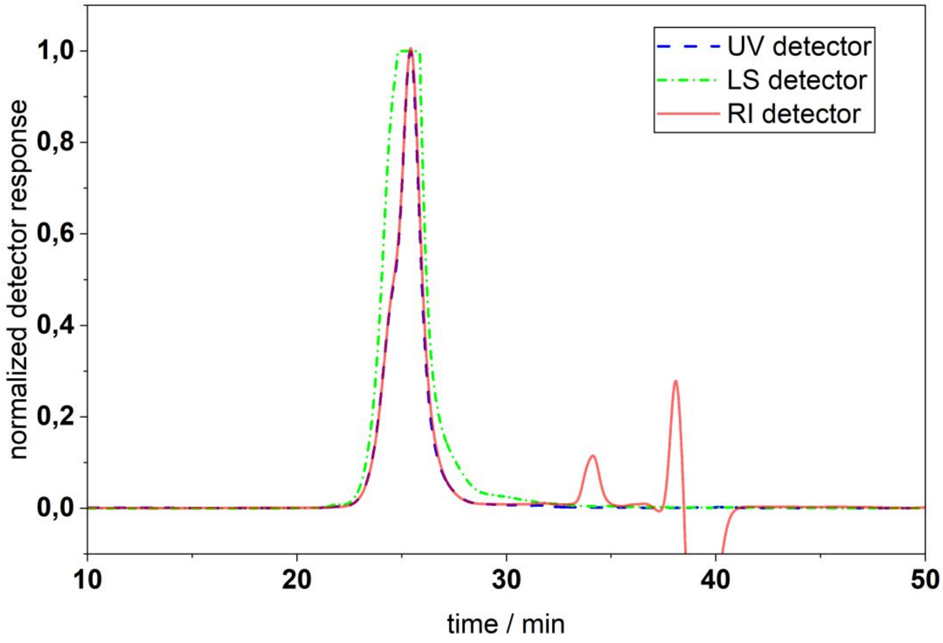

Figure S 11. SEC of Cy5-labelled PCPP in PBS pH 7.4.

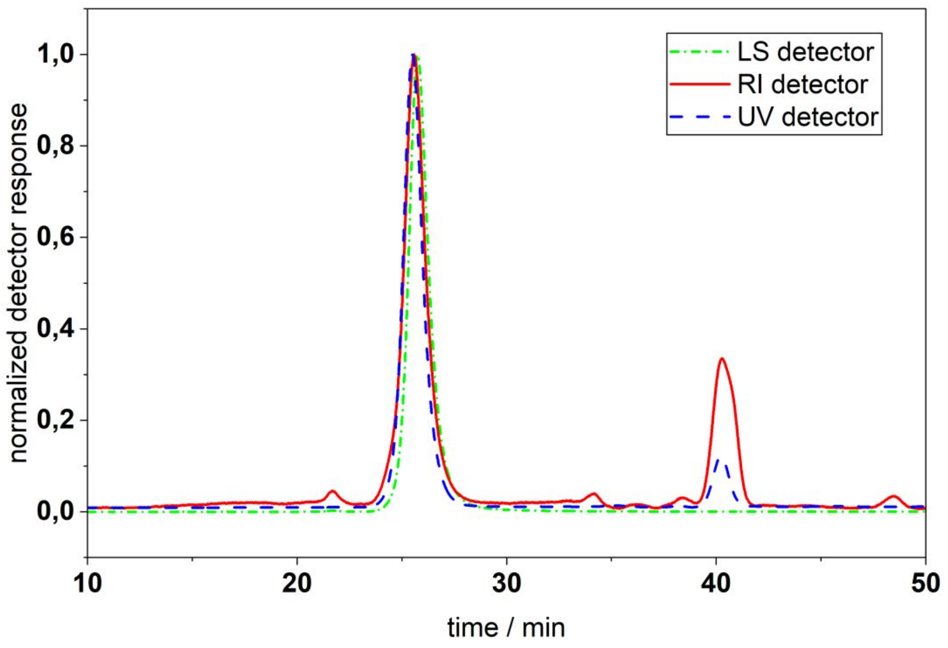

Figure S 12. SEC of Cy5-labelled PGA in Milli-Q-water.

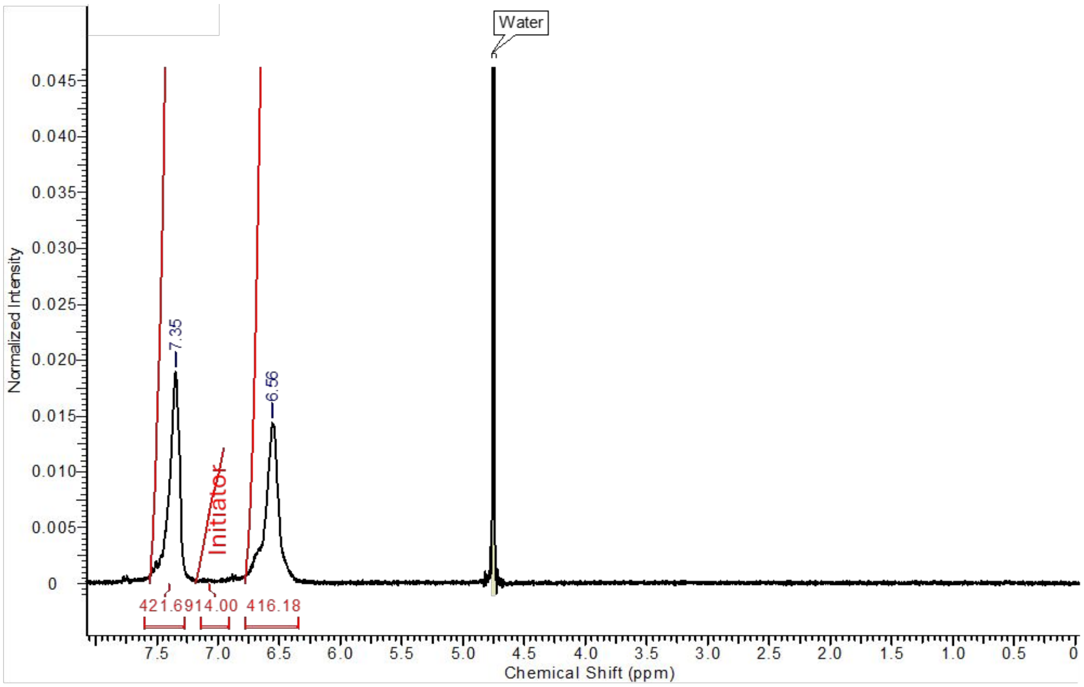

Figure S 13. ^{1}H-NMR (300 MHz) of PCPP in D_2O.

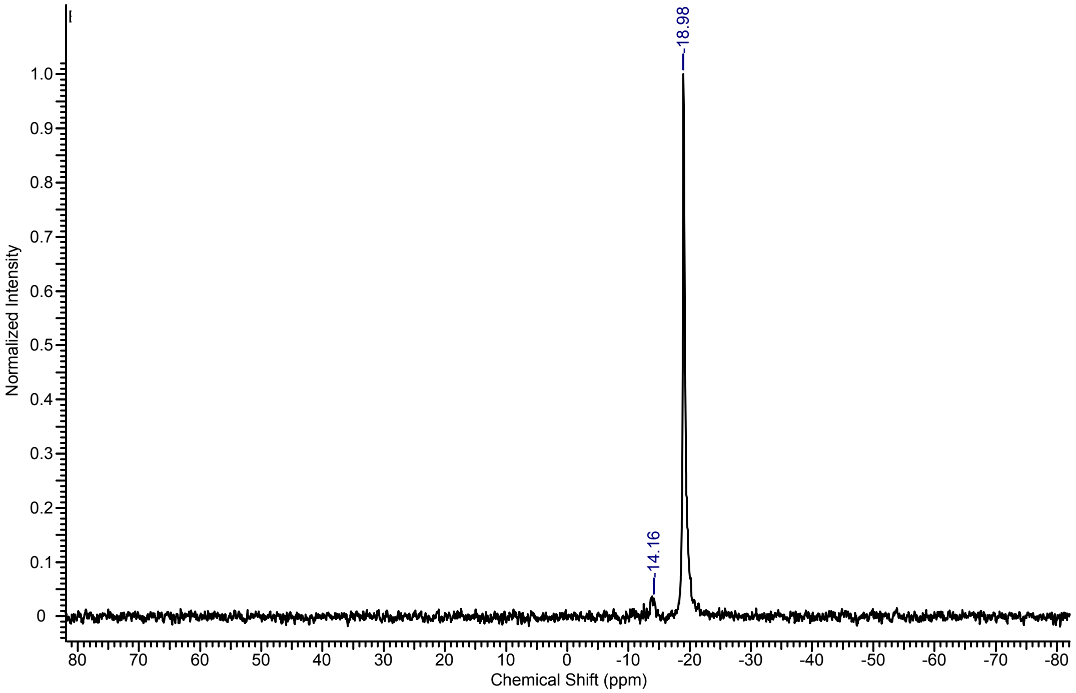

Figure S 14. ^{31}P-NMR (300 MHz) of PCPP in D_2O.

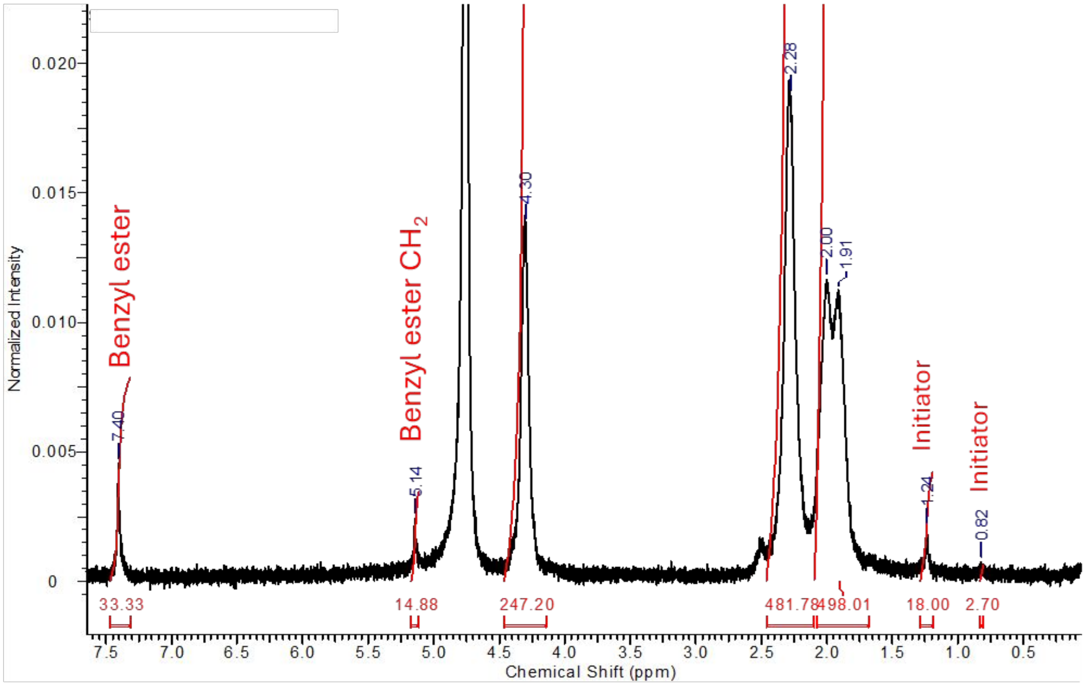

Figure S 15. ^{1}H-NMR (300 MHz) of PGA in D_2O.
